# Optimism Model by a Cardiology Pharmacist in Breaking Bad News Among Patients with CTRCD and its Impact on Outcomes

**DOI:** 10.5334/gh.1465

**Published:** 2025-09-02

**Authors:** Israa Fadhil Yaseen, Hasan Ali Farhan

**Affiliations:** 1 Cardio-Oncology Clinic, Baghdad Medical City, Baghdad, Iraq; 2 Scientific Council of Cardiology, Iraqi Board for Medical Specializations, Baghdad, Iraq; 3 College of Medicine, University of Baghdad, Baghdad, Iraq

**Keywords:** CTRCD, Cardio-Oncology, Outcomes, Breaking Bad News

Although breaking bad news is a painful truth, delivery is important. It could instill optimistic hope in the hearts of patients if delivered correctly, or it could cause an additional emotional wound for patients if delivered poorly. Bad news is not limited to death; it could be any news that affects a patient’s thoughts about his/her future (1). Successful communication skill is one of the keys for improving patient outcomes (2). Inappropriate communication with the patients may be the result of limited time (3).

Usually, physicians are responsible for breaking bad news. In a busy cardio-oncology clinic, cardiologists may not have the time to handle this delicate interaction while also providing medical care. Other healthcare professionals, such as pharmacists, can therefore assist them in breaking bad news (4). The role of pharmacist in the cardio-oncology clinic can help to maximise the time cardiologists have in clinic, allowing them more time to see a larger number of patients per clinic; among these roles is breaking bad news (5).

In 2015, a consultant cardiologist enrolled Iraq in the EURObservational Research Programme Peripartum Cardiomyopathy (EORP-PPCM) registry and involved a cardiology clinical pharmacist in the heart team to assist in breaking bad news in addition to other responsibilities (6). The cardiology pharmacist created a new model for breaking bad news called the Optimism Model that uses an optimistic statement before providing patients with the diagnosis of PPCM, leading to improvement in patients’ adherence to treatment and follow-up visits (5). In 2019, the consultant cardiologist and cardiology pharmacist founded the Iraqi Cardio-Oncology Program (ICOP), and then in 2020, they established the first cardio-oncology clinic in Iraq.

A sub-study related to the prospective observational ICOP registry was conducted to identify the impact of using the Optimism Model in breaking bad news on the adherence to treatment and follow-up visits and on the improvement in the left ventricular ejection fraction (LVEF) among patients with cancer who were newly diagnosed with cancer therapy-related cardiac dysfunction (CTRCD). The latter variables were already among the collected data in the main ICOP registry. Interventions by the cardiology pharmacist at the clinic were documented in the registry, including breaking bad news. The current study question and data analysis regarding the impact of the Optimism Model by a cardiology pharmacist on patients’ outcomes were set after completing two years of data collection. The study was approved by the National Research Ethics Committee. Patients were categorized into two groups (Central illustration 1-A).

**Central illustration 1:**
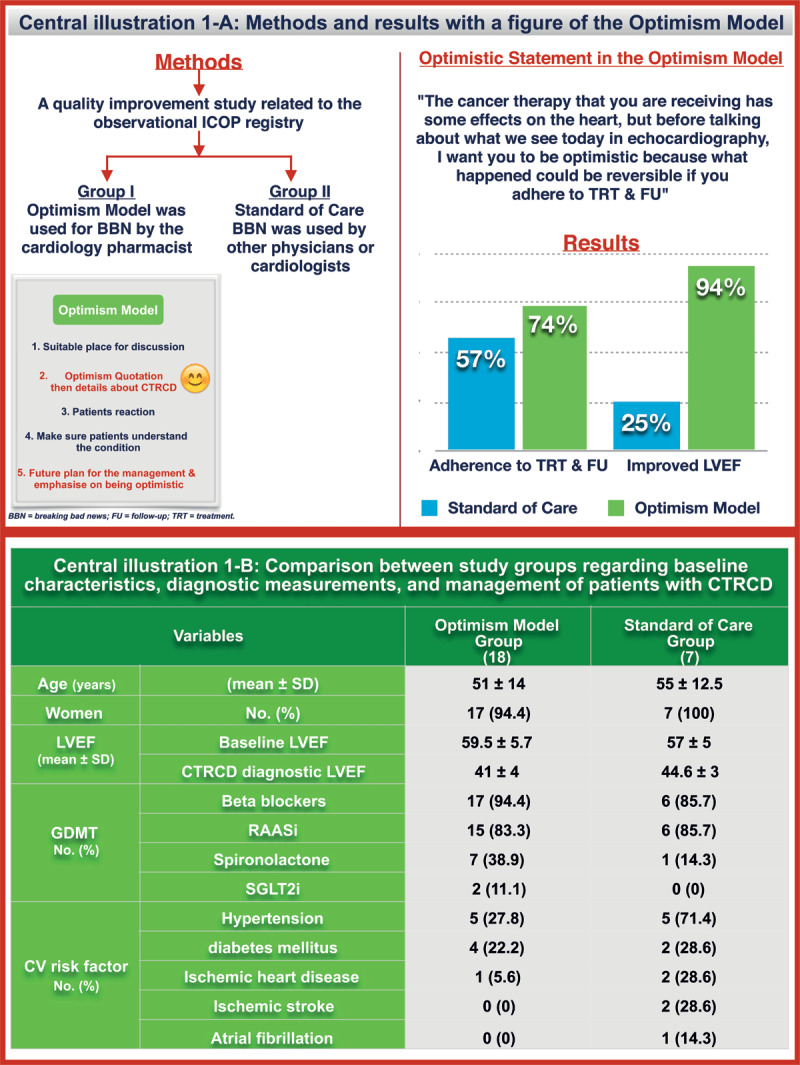
Methods, results, and the Optimism Model compared with the standard of care in breaking bad news among patients with CTRCD. CTRCD = cancer therapy-related cardiac dysfunction; CV = cardiovascular; GDMT = guideline-directed medical therapy; LVEF = left ventricular ejection fraction; RAASi = renin-angiotensin-aldosterone system inhibitor; SGLT2i = sodium-glucose cotransporter-2 inhibitor.

For group II, a standard of care model of breaking bad news was delivered by cardiology fellows in training at the cardio-oncology clinic when the cardiology pharmacist was on a vacation or by other physicians or cardiologists at other cardiology clinics, including private clinics.

Data was collected between December 2020 and December 2022. A total of 30 patients were included. Baseline characteristics, CTRCD diagnosis, and treatment were recorded (Central illustration 1-B). The most common type of cancer was breast 24 (80%). Of the total cardiovascular risk factors (CVRF) in both groups, 3 of 10 (30%) CVRF developed post-initiation of cancer therapy in group I, while 6 of 12 (50%) developed in group II. CTRCD was caused mainly by Trastuzumab 15 (50%) and 1 (3.3%) by anthracyclines; this patient was in group I and had complete LVEF recovery. The Optimism Model was delivered to 23 (77%) patients, with a loss of follow-up for five patients, resulting in 18 patients in group I. All patients attending the clinic with the diagnosis of CTRCD were included in the study with a follow-up period between 10 and 12 months. The cardiology pharmacist broke the bad news voluntarily. The Optimism Model, including the optimistic introductory statement and outcomes, is shown in the central illustration 1-A.

The current findings are not free of co-founders and limitations. First, because the study is observational, it lacks randomization and blinding. Also, since the leading author of the study created the Optimism Model, there is a possibility of bias in interpreting the study’s data. However, the sub-study design, which was not among the pre-specified aims of the main study, minimized bias and could not affect the outcomes documentation. Second, the higher rate of CVRF and the lower rate of prescribing treatments could contribute to the worse outcomes observed in group II. But at the same time, it is important to pay attention to the higher rate of CVRF developed post the initiation of cancer therapy in group II. Baseline CVRF theoretically increases worse outcomes of CTRCD because they are chronic and most likely developed over a long time before the diagnosis of cancer. On the other hand, cancer therapy-induced CVRF was triggered by a known underlying cause and developed over a short period, and it is reversible. Therefore, the impact of the latter CVRF is less likely to be the cause behind worse outcomes in group II. Also, it should be noted that the mean of LVEF at the diagnosis of CTRCD was lower in group I. This explains the higher rate of spironolactone and SGLT2i used in group I. Moreover, the low rate of SGLT2i use is interpreted by the time of the study, which was conducted in the period before the publication of updated international guidelines. Finally, this study reflects a real-world practice at a resource-limited setting in one of the low- to middle-income countries (LMICs). The intervention is novel and not associated with increased financial burden because it was delivered by the cardiology pharmacist voluntarily, and it is feasible to be applied at other LMICs.

In summary, findings showed that adherence of the patients with CTRCD to treatment and follow-up visits and improvement of LVEF were achieved in most of the patients for whom the Optimism Model was used, including the use of the optimistic introductory statement to draw patients’ attention to patient education, management, and follow-up. Breaking bad news with empathy is essential for better patient outcomes. A larger study and a clinical trial are required to confirm the results of the ICOP observational registry.
